# Tai Chi Expertise Classification in Older Adults Using Wrist Wearables and Machine Learning

**DOI:** 10.3390/s24154955

**Published:** 2024-07-31

**Authors:** Yang Hu, Mengyue Huang, Jonathan Cerna, Rachneet Kaur, Manuel E. Hernandez

**Affiliations:** 1 Department of Kinesiology, College of Health and Human Science, San José State University, San Jose, CA 95129, USA; yang.hu@sjsu.edu; 2 School of Information Sciences, University of Illinois Urbana-Champaign, Urbana, IL 61801, USA; mengyue4@illinois.edu; 3 Neuroscience Program, University of Illinois Urbana-Champaign, Urbana, IL 61801, USA; cerna3@illinois.edu; 4 Department of Industrial and Enterprise Systems Engineering, University of Illinois at Urbana-Champaign, Urbana, IL 61801, USA; rk4@illinois.edu; 5 Department of Biomedical and Translational Sciences, University of Illinois Urbana-Champaign, Urbana, IL 61801, USA; 6 Department of Bioengineering, University of Illinois Urbana-Champaign, Urbana, IL 61801, USA; 7 Department of Kinesiology and Community Health, University of Illinois Urbana-Champaign, Urbana, IL 61801, USA; 8 Beckman Institute, University of Illinois Urbana-Champaign, Urbana, IL 61801, USA

**Keywords:** machine learning, Parkinson’s disease, wearables

## Abstract

Tai Chi is a Chinese martial art that provides an adaptive and accessible exercise for older adults with varying functional capacity. While Tai Chi is widely recommended for its physical benefits, wider adoption in at-home practice presents challenges for practitioners, as limited feedback may hamper learning. This study examined the feasibility of using a wearable sensor, combined with machine learning (ML) approaches, to automatically and objectively classify Tai Chi expertise. We hypothesized that the combination of wrist acceleration profiles with ML approaches would be able to accurately classify practitioners’ Tai Chi expertise levels. Twelve older active Tai Chi practitioners were recruited for this study. The self-reported lifetime practice hours were used to identify subjects in low, medium, or highly experienced groups. Using 15 acceleration-derived features from a wearable sensor during a self-guided Tai Chi movement and 8 ML architectures, we found multiclass classification performance to range from 0.73 to 0.97 in accuracy and F1-score. Based on feature importance analysis, the top three features were found to each result in a 16–19% performance drop in accuracy. These findings suggest that wrist-wearable-based ML models may accurately classify practice-related changes in movement patterns, which may be helpful in quantifying progress in at-home exercises.

## 1. Introduction

Tai Chi (or Tai Ji Quan, TC) is a Chinese martial art that combines physical practice, breathing techniques, and meditation; it is accessible and does not have a high base physical functioning requirement for practitioners. TC practice requires no equipment and little space, and it can be practiced anytime, anywhere, and by older adults and individuals with chronic conditions [1]. Moreover, a systematic review found that TC could be performed by those with chronic medical conditions, such as chronic obstructive pulmonary disease, heart failure, and osteoarthritis, without worsening shortness of breath and pain, and found favorable effects on functional exercise capacity in people with these conditions [2]. Furthermore, TC practice also has been widely used as a nonpharmacological approach to improve postural control and prevent falls in older adults [3]. In 2015, the Centers for Disease Control and Prevention (CDC) recommended TC as an exercise program for reducing falls in older adults [4] and showcased some simplified TC programs that improve balance and reduce fall risk in older adults, such as “Tai Chi: Moving for Better Balance” [5].

In traditional TC training, practitioners receive valuable in-person feedback from trainers, encompassing cues related to movement patterns, speed, mindfulness, and breathing techniques—all crucial aspects of the practice [6]. However, with the growing popularity of TC and the inherent limitations of in-person instruction, accessing consistent and individualized feedback becomes increasingly challenging for practitioners. This gap necessitates exploring innovative methods for self-assessment and self-improvement, particularly in the physical and cognitive domains of TC.

Indeed, the issue of assessing proficiency or expertise in mind–body practices with a movement component such as TC, Qigong, or yoga remains a significant challenge for several reasons. Such challenges include, but are not limited to, a lack of standardized assessment tools, the inherent subjectivity in evaluation of movement quality and form, and the multidimensional nature (i.e., involvement of physical and mental components) of the practice, which makes it challenging to capture all aspects of proficiency through a single measure, among other challenges.

Advances in technology may offer potential solutions to bridge this gap and enhance the learning experience for TC practitioners. Among such advancements, inertial measurement units (IMUs) and accelerometry-based wearable devices provide an avenue to provide individualized and timely feedback regarding movement patterns, form, and speed. These wearable devices tend to be more cost-permissive, smaller in dimensions, and lightweight as compared to the standard laboratory equipment used for kinematic and kinetic parameterization. Consequently, researchers have implemented them in various clinical settings (e.g., measuring postural abnormalities in Parkinson’s Disease via stabilometry) and daily life activity assessments (e.g., tremor assessment via accelerometry) (see Iosa et al., 2016 [7] for more details). In addition, technology, such as virtual reality (VR) and augmented reality (AR) have been used to augment or broaden participation in Tai Chi practice [8,9,10]. While the potential of wearable sensors for providing objective feedback in TC training is promising, to date, no study has investigated their ability to establish a benchmark or identify objective behavioral phenotypes indicative of TC proficiency, which may be helpful for fine-tuning VR/AR practice recommendations.

While TC is a full-body exercise, it also emphasizes the control of arms and hands [11,12]. Previous studies have suggested that wrist acceleration data are valid for describing the control of the arms and hands [13,14]. Moreover, many adapted and modified TC practices recommended for older populations or people who lack mobility are focused on upper body movement, such as seated TC [15] or wheelchair Tai Chi [16]. Thus, developing machine learning models based only on data collected from the upper body is relevant and valuable in accommodating all different types of practitioners.

In this project, we investigated the feasibility of using a single wrist-worn wearable sensor to classify the expertise of older adults with different TC experience levels. We hypothesized that the combination of wrist acceleration profiles during a well-rehearsed movement together with ML approaches would be able to accurately classify practitioners’ TC expertise levels. Further, we evaluated the importance of acceleration-derived features in the top three performing ML architectures, and the ability to generalize to unseen TC practitioners.

## 2. Materials and Methods

### 2.1. Protocol

The Institutional Review Board approved the protocol for the study under IRB number 15317.

During the lab visit, the TC practitioner was asked to perform five sets of Cloud Hands at a self-selected speed and form. The form and speed were not specifically instructed, but each set was required to include the start form, be repeated at least three times to the left, repeated at least three times to the right, and also include the end form. The participants were instructed to take breaks between each set if needed. Acceleration data were collected with a wireless wearable wristband (E4 wristband, Empatica Inc., Cambridge, MA, USA) placed on the right wrist before the start of the practice. This task took approximately 5 to 15 min to be administered.

### 2.2. Participants

We recruited twelve older adult TC practitioners from the local community to participate in this study. The self-reported continued practice hours were used to assess the TC experience levels and categorize them into less experienced, moderately experienced, and highly experienced groups. The cutoffs for experience levels (<1560 h, 1560–2496 h, and >2496 h, respectively) were determined based on the distribution of self-reported practice hours among the participants, representing natural breaks in the data. Based on this benchmark, three participants were grouped into less experienced (one male, age: 73–79 years, practice hours: 61–234 h), five participants were grouped into moderately experienced (two males, age: 65–82 years, practice hours: 1560–1872 h), and four participants were grouped into highly experienced groups (one male, age: 68–87 years, practice hours: 2496–4680 h), respectively. It is also worth noting that all practitioners came from two local TC groups and practiced in a similar style (i.e., Yang style 24 form or modified Yang style).

### 2.3. Data Preprocessing

In order to attenuate noise in the signals, accelerometer data were filtered using a bandpass Butterworth filter with 0.1 Hz high-pass and 30 Hz low-pass cutoffs (Equation (1)).

(1) $$H_{\left(j\omega \right)}=\frac{1}{\sqrt{1+\epsilon^{2}{\left(\frac{w}{w_{p}}\right)}^{8}}}$$

Following filtering, the resulting jerk $J_{\left(t\right)}$ was calculated using the following equation (Equation (2)) and plotted for visual inspection.

(2) $$J_{\left(t\right)}=\sqrt{{\frac{dx}{dt}}^{2}+{\frac{dy}{dt}}^{2}+{\frac{dz}{dt}}^{2}}$$

where $\frac{dx}{dt}$, $\frac{dy}{dt}$, and $\frac{dz}{dt}$ are the first time derivative of the raw acceleration signal in *x*-, *y*-, and *z*-directions, and, thus, the calculated jerk in *x*-, *y*-, and *z*-directions, respectively.

**Figure 1:**
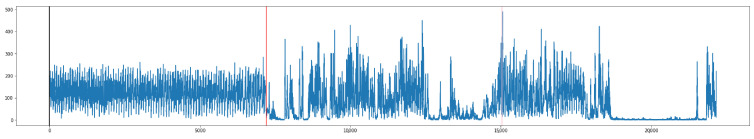
An example data profile of calculated jerk from one participant. Note: The black line marks the identified beginning of the cloud hand practice; the red line marks the identified end of the cloud hand practice.

Based on the resulting jerk profile and the periodic nature of cloud hand movement, the beginning and end of the cloud hand practice were identified and labeled for data segmentation and feature extraction. Figure 1 illustrates an example resultant jerk profile of the participant.

### 2.4. Data Segmentation and Feature Extraction

**Figure 2:**
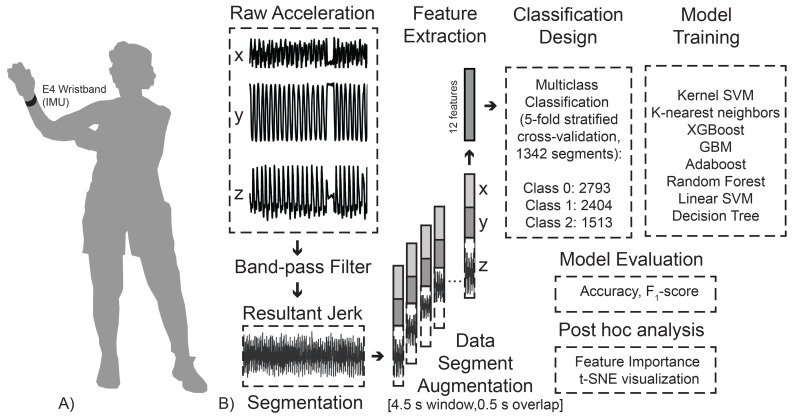
(**A**) Experimental setup and (**B**) workflow pipeline.

Once the cloud hand practice task was segmented, data segment augmentation was carried out using a 4.5 s (144 sample) window with a 0.5 s (16 sample) overlap, which resulted in a total of 2793 segments for the less experienced group (Class 0), 2404 segments for the moderately experienced group (Class 1), and 1513 segments for the highly experienced group (Class 2) (see Figure 2). Within each data segment, raw triaxial acceleration from the E4 wristband were collected.

Acceleration data were collected from a single accelerometer using raw mode and were transformed into g-units. For each time point, the resultant acceleration ($r_{i}$) was calculated as follows:

(3) $$r_{i}=\sqrt{x_{i}^{2}+y_{i}^{2}+z_{i}^{2}}$$

where $x_{i}$, $y_{i}$, and $z_{i}$ are the *i*th measurement sample of the raw acceleration signal in *x*-, *y*-, and *z*-directions.

**Figure 3:**
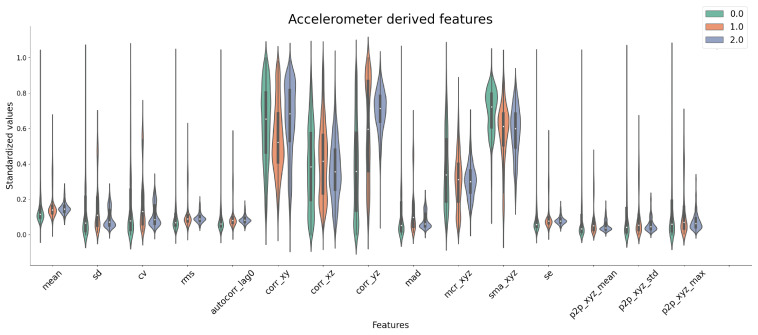
Distribution patterns of each feature across all 3 classes of Tai Chi expertise. NOTES: Class 0 = less experienced group; Class 1 = moderately experienced group; Class 2 = highly experienced group.

Traditional characteristics of recorded periodic movement accelerations, such as the mean (μ), standard deviation (σ), coefficient of variance (CV), root mean square (RMS), autocorrelation (*A*), mean amplitude deviation (Equation (4)) [17] (MAD), signal magnitude area (Equation (5)) (SMA), signal energy (area under the squared magnitude) (SE), paired correlation coefficient ($r_{xy},r_{yz},r_{xz}$), peak-to-peak mean ($\mu^{P2P}$), standard deviation ($\sigma^{P2P}$), and maximum ($\gamma^{P2P}$) were examined in the accelerometer sensor placed on the wrist for differences in older Tai Chi practitioners in different experience groups. Figure 3 compares the distributions of accelerometer-derived features between subjects in different groups.

(4) $$MAD=\frac{1}{n}\sum \left|r_{i}-\bar{r}\right|$$

(5) $$SMA=\frac{1}{T}\int_{0}^{T}(|x\left(t\right)-\bar{x}|\;+\;|y\left(t\right)-\bar{y}|\;+\;|z\left(t\right)\;-\;\bar{z}\left|\right)dt$$

### 2.5. Model Selection, Training, and Evaluation

Before diving into modeling, a data structure exploration with t-SNE plot was conducted. t-Distributed Stochastic Neighbor Embedding (t-SNE) is a powerful dimensionality reduction technique commonly used for visualizing high-dimensional data. t-SNE helps in reducing data dimensions to two or three, making it feasible to plot and visually inspect the data; thus, it could effectively group similar data points together, which makes room for identification of clusters or classes within the data.

For the modeling process, the performance of eight state-of-the-art supervised learning classifiers—kernel support vector machine (SVM), K-nearest neighbor (KNN), extreme gradient boosting (XGBoost), gradient boosting machine (GBM), adaptive boosting (Adaboost), random forest, and linear SVM—was compared using features standardized with its own distribution. To address the potential issues in limited dataset size, imbalanced labels, and tuning efficiency, 5-fold cross-validation (CV) with StratifiedKFold was employed while training. This approach involved training the algorithms on randomly selected five parts, each containing 4.5-second signals from four parts of training trials, and validating on the fifth part, which contained 4.5-second signals from the remaining trials. This process was repeated five times until all five subsets were utilized for validation from the data. Due to the imbalanced nature of the class labels (0, 1, 2), StratifiedKFold is a desired CV method here for its ability to maintain label composition similarity across each fold during conducting CV.

In terms of insight gaining from models, their confusion matrix, performance metrics, and permutation importance was found. For those analyses, a train–test split with 0.2 test size and 0 random state from sklearn was utilized. Permutation importance was utilized to understand each variables’ predictive power brought to the model and the general relationship between movement and TC levels. We evaluated the performance of the models on the basis of accuracy, precision, recall, and $F_{1}$-score, all unweighted (micro), placing equal importance on each label.

The implementation of analysis was carried out using Python (version: 3.10.13) via open-source libraries such as sklearn and pandas.

## 3. Results

Considering a formal comparison of differences of each feature across all three classes of TC expertise (Figure 3), we find no statistically significant differences, based on a nonparametric test of group differences using the Kruskall–Wallis test. These findings further motivate the use of ML for identifying TC expertise differences.

**Figure 4:**
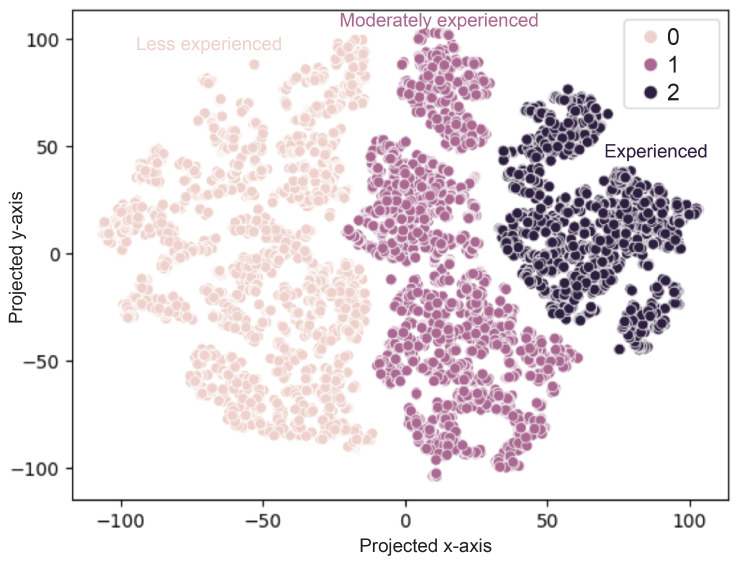
t-SNE visualization of three classes of TC practitioners.

For the t-SNE plot (Figure 4), a plot with perplexity value of 10 showed the potential clustering structure of the data, balancing the attention between local and global aspects of the data structure. The clusters were well separated, indicating an inherent separated structure of the data among different labels, allowing for clear differentiation between the three classes.

For the models, it is observed that a consistent high overall performance occurs across various label-wise models for distinct metrics (Table 1). The lowest accuracy figure recorded was approximately 72.3%, while the majority of models achieved accuracy levels above 90%. The consistency in the decent performance across models shows the predictive power from our TC movement data.

**Figure 5:**
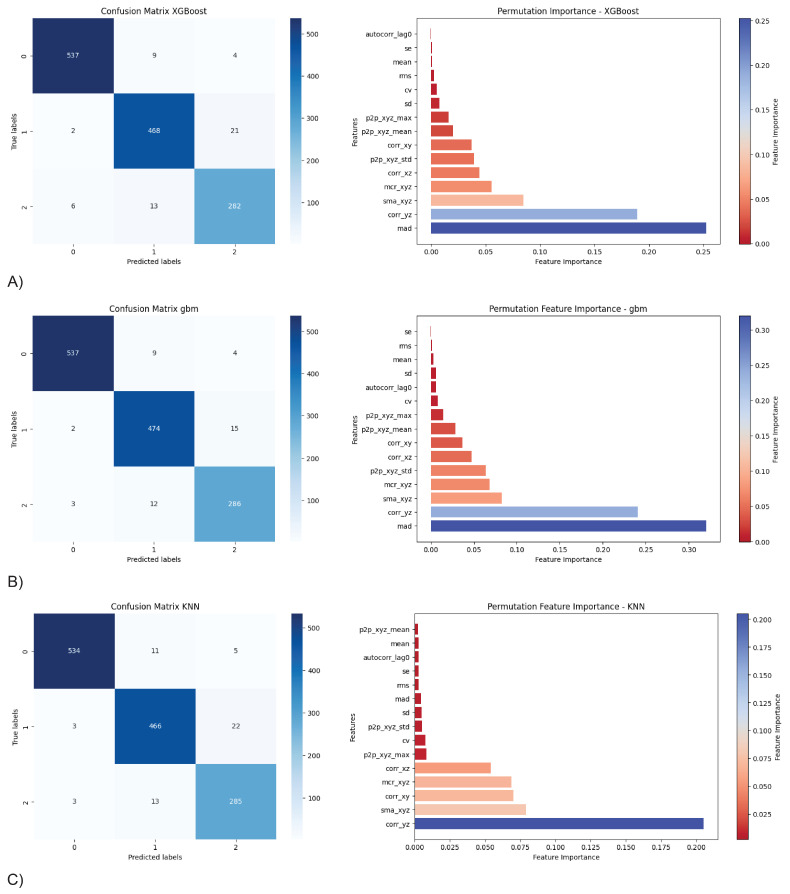
Model performance and feature importance of the top 3 classification models: (**A**) XGBoost, (**B**) GBM, and (**C**) KNN.

**Figure 6:**
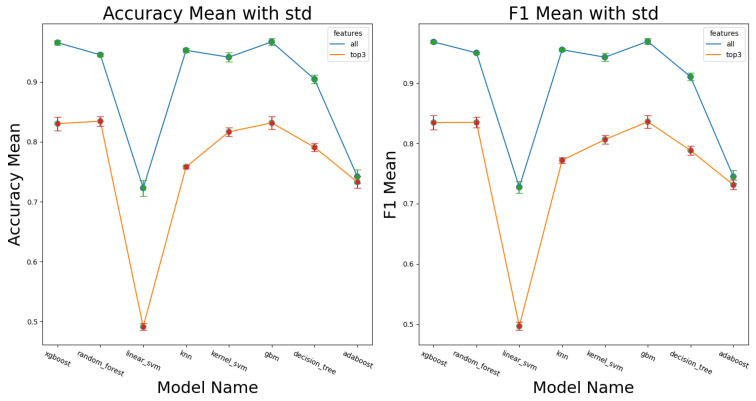
Differences in performance between full model and use of model with only the top 3 features for all ML architectures.

In terms of feature importance, a consensus among the models was found regarding the most influential features contributing to the model’s performance (Figure 5). Specifically, MAD, $r_{yz}$, and SMA are the most crucial features across most of the models. The accuracy for the top three models (XGBoost, GBM, KNN) using only their top three features decreased by 13.4% (from 96.5% to 83.1%), 13.5% (from 96.7% to 83.2%), and 19.4% (from 95.2% to 75.8%), respectively. This indicates that the top features in these models contributed greatly to the effectiveness of these classifiers, as seen by small drop-off in accuracy or F1 classification performance when using the top three features (Figure 6).

With our labels from the original data being imbalanced with less experienced practitioners having 2793 instances, moderately experienced practitioners having 2404 instances, and highly experienced practitioners having 1513 instances (42%, 36%, and 23% of total data), the top three models of XGBoost, GBM, and KNN all showed relatively more difficulty when trying to distinguish between label1 and label2 in their confusion matrix (Figure 5), despite their great overall performance figure above 95.2% on accuracy. The models, while achieving high overall accuracy (above 95.2%), struggled to distinguish between moderately experienced practitioners and highly experienced practitioners. This is likely due to the data themselves, where the gap in participants’ lifetime practice hours is smaller between these two practitioner groups as compared to moderately experienced and less experienced practitioners. The proportion of misclassifications specifically between moderately experienced practitioners and highly experienced practitioners was significant: 54.76% for XGBoost, 57.14% for GBM, and 61.40% for KNN.

## 4. Discussion

Overall, this study used a single wrist-worn wearable sensor and supervised learning classifiers to classify the three-way expertise of older adults with different TC experience levels. Our results suggest that this approach is feasible and yields decent to high performance across selected models.

Our results contribute to the existing literature in several significant ways. Previous studies have provided detailed biomechanical analyses of TC practice [18,19,20], yet none have integrated such kinematic or kinetic data into machine learning (ML)-enhanced classification models. The identification of key features, particularly those related to MAD and SMA, underscores the biomechanical underpinnings of TC proficiency. These features likely enable ML models to capture the essential elements of fluidity, balance, and control that are central to TC practice. Our results suggest that MAD and SMA are the most crucial features across most models, supported by sustained high performance (>75% classification accuracy despite several variables being dropped) in our label-wise tuned models. This finding aligns with the traditional teachings of TC and the current understanding of its biomechanical characteristics.

MAD describes the acceleration amplitude deviation from the average acceleration of the movement, quantifies movement variability, and reflects how much the movement pattern deviates from the average pattern [17]. Conceptually, the mean amplitude deviation is similar to the mean absolute deviation in math, and, generally, the higher the MAD, the higher the variability of the periodic movement. From our understanding, no previous studies used MAD to describe the movement variability in Tai Chi practice. However, previous studies have demonstrated that the practice of Tai Chi reduced upper limb force variability [21]. SMA is the normalized integral of the original acceleration value. It is a statistical measure of the magnitude of the varying acceleration quantity and quantifies the overall magnitude of the signal over a specified time period [22]. Conceptually, SMA is similar to the root mean square. To our understanding, no previous studies have used SMA in studies regarding Tai Chi practice. However, previous studies have used the root mean square to investigate the Tai Chi practice-related changes in center of pressure [23,24], muscle activation [25], and more.

The use of the Cloud Hands TC form, also be known as “wave hands like clouds” or “Yun Shou”, in this study provides the examination of a common practice form in most TC styles. Thus, this study provides a TC-practice-specific paradigm rather than an occupational task such as holding a full cup of water. By training ML models with TC-specific movement data, instead of other occupational tasks, this model demonstrates the potential to objectively assess TC practice progress.

These findings not only extend the scope of the existing literature into practical applications but also open the door to leveraging other physiological and neurophysiological modalities into advanced classification schemes. Previous evidence [26] shows that visceral signals dynamically interplay and actively shape large-scale neural dynamics. Given their interplay, information derived from such physiological (e.g., cardiac, respiratory, and kinematic data) and neurophysiological (e.g., dynamic functional connectivity from EEG, fNIRS, or classically fMRI) sources should be paired in future work to attempt to capture proficiency in a more holistic fashion. By integrating these multimodal data, future research can develop more comprehensive and nuanced models of expertise. Ultimately, the goal would be for such schemes to provide practitioners with detailed, actionable feedback to facilitate and accelerate learning.

Compared to traditional feedback and rating systems, which are often subjective and prone to inter-rater variability, this approach provides an objective and quantifiable measure of expertise. The use of wearable sensors allows for continuous data collection and analysis, providing a more comprehensive and detailed understanding of movement patterns than is possible with traditional observation-based assessments. Furthermore, ML algorithms can identify subtle variations in movement that may not be readily apparent to human observers, enabling a more nuanced evaluation of proficiency. While this approach holds promise for providing more accessible and scalable feedback compared to traditional methods, further research is needed to evaluate its feasibility, as well as its reception and adoption among those interested in engaging in deliberate practice.

Despite the strengths of the study, various limitations should be pointed out. First, our small sample and lack of a control group limit generalizability and validation of TC-practice expertise specificity. Further, our categories of expertise relied on self-reported lifetime practice hours. This method has issues related to imperfect recall [27,28], social desirability [29,30], and the assumption that practice hours equate to proficiency levels. Although recent dose–response studies in mind–body practice (with research primarily limited to meditation practice) are starting to corroborate the relationship between practice hours, cognitive, and mental health benefits [31], most research on mind–body practices still lacks granularity and tacitly assumes that hours of practice are equivalent to a certain level of proficiency [32]. Further research should explore this assumption via alternative classification schemes, potentially integrating objective performance measures, peer assessments, and self-report.

In addition, this study highlights common issues that pose limitations for generalizability. Namely, TC practice classification due to the low heterogeneity in practice forms (i.e., data derived solely from cloud hands) practitioners engaged in, as well as practice styles among participants, allows for more accurate modeling of expertise levels using ML algorithms. On one hand, the consistency of practice styles among participants allows for more accurate modeling of expertise levels using ML algorithms, as seen in the high classification performance across different models. This homogeneity ensures that the ML models are not confounded by variations in TC styles, thus enhancing the internal validity of the study. However, this also limits the external validity, as the findings may not be readily applicable to other forms or variations of TC, such as those with differing kinetic and kinematic characteristics (e.g., Wu, Sun, Hao, Chen). Future research should aim to incorporate a broader range of Tai Chi forms and styles to improve the generalizability of the ML models and enable the discernment between different styles while maintaining the ability to categorize based on proficiency levels.

Furthermore, understanding the coupling and relationships between different metrics is crucial for deepening our knowledge of the connection between objective markers and expertise in mind–body practices such as TC, yoga, and meditation. For instance, the interplay between cardiac and neural responses, or the synchronization of respiratory and movement patterns [26], can provide comprehensive insights into the physiological and cognitive dimensions of these practices. As mentioned previously, such integration of metrics could enhance the precision of ML models in classifying expertise by capturing the multidimensional nature of proficiency in these disciplines. Incorporating these relationships into future studies could pave the way for more holistic and accurate assessments of expertise in mind–body practices, facilitating better feedback and learning outcomes for practitioners.

## 5. Conclusions

In conclusion, this study demonstrates the potential of using wearable sensors combined with supervised learning classifiers to objectively measure and classify TC proficiency among older adults. The consistent high performance of our models, even with the inherent imbalances in the dataset, highlights the robustness of this approach. Our approach highlights early attempts to leverage the capacity of ML algorithms to effectively differentiate between varying levels of TC expertise, particularly through the identification of key features such as MAD and SMA. These features may be capturing essential aspects of movement such as fluidity, balance, and control, which are crucial to TC practice.

However, this study also identifies several areas for further research and improvement. The homogeneity in practice type, as well as self-reported practice hours as a measure of proficiency, introduces potential biases and limits the external validity of the classification models. Future studies should explore alternative and more objective measures of proficiency, incorporating peer assessments and direct performance metrics to discern between proficiency levels. Additionally, expanding the study to include a broader range of TC styles could improve the generalizability of the models and enhance their applicability across different forms of practice. By integrating multimodal data, including physiological and neurophysiological metrics, future research can develop more comprehensive and nuanced models of expertise, ultimately providing practitioners with detailed and actionable feedback to facilitate learning and improvement.

## Figures and Tables

**Table 1:** Model performance metrics.

Model	Accuracy	F1
gbm	0.9666±0.0057	0.9692±0.0049
xgboost	0.9652±0.0038	0.9683±0.0028
knn	0.9524±0.0039	0.9553±0.0024
random forest	0.9448±0.0027	0.9501±0.0017
kernel svm	0.9409±0.0079	0.9429±0.0070
decision tree	0.9043±0.0070	0.9104±0.0063
adaboost	0.7418±0.0112	0.7447±0.0110
linear svm	0.7227±0.0132	0.7273±0.0096

## Data Availability

The data that support the findings of this study are available on request from the corresponding author, M.E.H.

## Abbreviations

The following abbreviations are used in this manuscript:

TC	Tai Chi
IMU	Inertial Measurement Unit
